# Alterations of Regional Homogeneity in Crohn's Disease With Psychological Disorders: A Resting-State fMRI Study

**DOI:** 10.3389/fneur.2022.817556

**Published:** 2022-02-02

**Authors:** Mengting Huang, Xin Li, Wenliang Fan, Jing Li, Liangru Zhu, Ping Lei, Linxia Wu, Qing Sun, Yan Zou, Ping Han

**Affiliations:** ^1^Department of Radiology, Tongji Medical College, Union Hospital, Huazhong University of Science and Technology, Wuhan, China; ^2^Hubei Province Key Laboratory of Molecular Imaging, Wuhan, China; ^3^Division of Gastroenterology, Tongji Medical College, Union Hospital, Huazhong University of Science and Technology, Wuhan, China

**Keywords:** Crohn's disease, regional homogeneity, resting-state functional MRI, brain function, psychological disorders

## Abstract

Abnormal psychological processing in the central nervous system has been found in Crohn's disease (CD) patients. Resting-state functional magnetic resonance images of 57 inactive and 58 active CD patients, and 92 healthy controls (HC) were obtained. The psychological assessment used a psychological questionnaire that was collected within 1 week before functional MRI examination. We investigated the neural basis of CD patients and the correlation among regional homogeneity (ReHo), clinical features and psychological assessment scores. We found that more severe psychological assessment disorder scores were observed in the active CD group than in the inactive CD group and HC group (*P*<0.001). Compared with HC, the active CD patients exhibited higher ReHo values in the frontal superior medial, frontal middle and lower values in the postcentral, supplementary motor area, and temporal middle. Meanwhile, inactive CD patients exhibited higher ReHo values in the frontal middle and lower ReHo values in the precentral, postcentral and putamen (all voxel P< 0.001, cluster P<0.01, corrected). The values of the frontal superior medial, postcentral and supplementary motor area were correlation with psychological assessment scores (r = 0.38, −0.41, −0.32, *P* = 0.001, 0.014, 0.003), and the clinical features of simple endoscopic score for Crohn's disease and erythrocyte sedimentation rate were negatively correlated with psychological assessment scores in active CD patients (r = −0.35, −0.34, *P* = 0.06, 0.08). These results provide evidence for abnormal resting-state brain activity in CD and suggest that the psychological of CD may play a critical role in brain function.

## Introduction

Crohn's disease (CD) is an autoimmune and relapsing gastrointestinal disease. The etiology remains unclear. The clinical manifestations are diverse, including gastrointestinal, systemic, and extraintestinal manifestations and complications such as diarrhea, abdominal pain, fatigue, fistula, and abdominal abscess. CD occurs mainly in young adults (1–3). Recent reports have shown that the incidence of CD is increasing rapidly in Asia, especially in China (4–6). Moreover, CD patients typically require lifelong medication, which seriously affects their quality of life and increases psychological distress (7).

Studies have shown that gray matter structure changes and white matter lesions may be the extraintestinal manifestations of CD that may induce psychological disturbances (8, 9). However, the pathogenesis remains unclear. The brain-gut axis plays a significant role in maintaining gastrointestinal homeostasis through regulation of the brain, cognitive functions and emotion. Through the brain-gut axis, gastrointestinal inflammatory signals are transmitted to the brain, causing the structural reorganization of neurotransmitters. In turn, the brain can regulate the intestinal tract in the same way, which presumably includes the limbic system, hypothalamus and cerebral cortex area (10). It has been reported that 34.7% of patients with active CD and 19.9% of patients with inactive CD were found to have anxiety and depression (11, 12). More anxiety and depression were found in CD patients than in healthy controls, which may exacerbate the effects of the disease. Furthermore, the intestinal microbiome is likely to interact with the brain.

Resting-state functional magnetic resonance imaging (rs-fMRI) can detect blood oxygenation level-dependent (BOLD) signals and explore the processing of various input signals in regions of the brain. Recently, fMRI has been used to study changes in brain gray matter structures in irritable bowel syndrome, ulcerative colitis and CD. Regional homogeneity (ReHo) is another analytical method that displays the synchronization of fluctuations among adjacent voxels of BOLD to provide information about local activity (13).

Currently, only a few studies have examined brain activity in patients with CD. The sample size was small, and the conclusions were not entirely consistent. In a study involving 25 CD patients with and without abdominal pain and 32 healthy controls (HCs), abnormal activities in the insula and middle cingulate cortex were closely related to the severity of abdominal pain (14). Another study by this research group identified no difference in those brain regions between CD patients and HCs (15, 16). Therefore, further investigations are necessary to determine whether changes exist in specific brain regions in CD patients and whether these changes are correlated with gastrointestinal disease activity and psychological symptoms.

However, few studies have examined ReHo in patients with active CD patients. Therefore, in this study, we divided CD patients into an active CD group and an inactive CD group and employed ReHo to compare the differences in aberrant brain activity between the two CD patient groups and the HC group. We hypothesized that CD patients would have altered aberrant brain function and that brain neuroimaging of ReHo may be correlated with clinical features and psychological assessment scores.

## Materials and Methods

This research was approved by the institutional ethics committee of Tongji Medical College of Huazhong University of Science and Technology, and acquired the consents of all participants (Protocol Number ICH S016). All subjects signed informed consent forms.

### Participants

A total of 129 CD patients were recruited through continuous unselected recruitment between December 2019 and July 2021 at the IBD specialist outpatient clinic of the Endoscopy Center of Union Hospital, Tongji Medical College, Huazhong University of Science and Technology in China. All patients received endoscopy, pathology biopsy and hematological examination and were evaluated by an experienced gastroenterologist (L.R.Z) within 2 weeks before MRI. The simple endoscopic score for Crohn's disease (SES-CD), Crohn's disease activity index (CDAI), erythrocyte sedimentation rate (ESR), C-reactive protein (CRP), platelet (PLT) levels and disease duration in years were collected from all patients (17, 18).

Inclusion criteria were as follows: (a) diagnosed with CD; (b) right-handed; (c) age 18 to 60 years; (d) education level more than 9 years; and (e) native Chinese speaker. The exclusion criteria were as follows: (a) patients with claustrophobia or metal implants; (b) patients who had received CD-related abdominal surgery; (c) pregnant or lactating women; (d) patients who used psychotropic drugs or opioids in the past 3 months; and (e) patients with current or a history of head trauma, tumor or loss of consciousness. Psychiatric examinations were assessed by a psychiatrist based on a psychiatric interview tool from the Diagnostic and Statistical Manual of Mental Disorders, Fifth Edition (DSM-5). The inactive CD group included patients with CD who had CDAI scores ≤150. The active CD group included patients with CD who had CDAI scores >150.

**Figure 1:**
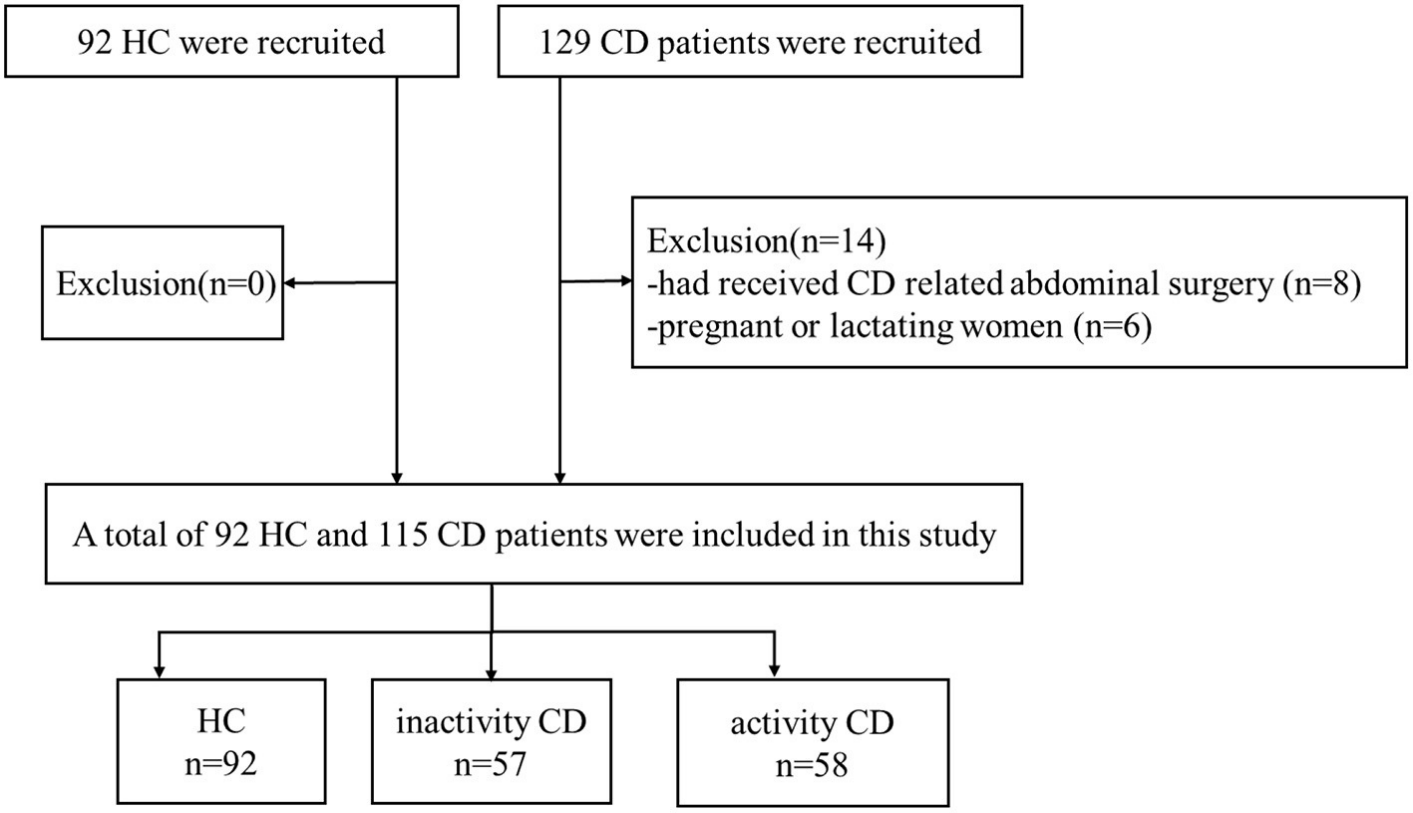
Flow diagram of the enrolled patients.

A total of 92 HC who were recruited by advertisements and matched with CD patients by age, sex, handedness, and education level as much as possible were included. The following exclusion criteria for HC were as follows: (a) patients with claustrophobia or metal implants; (b) pregnant or lactating women. The flow diagram of the enrolled CD patients and HC is shown in Figure 1.

### Clinical Features and Psychological Assessment

The CDAI, SES-CD, laboratory tests, Inflammatory Bowel Disease Questionnaire (IBDQ), Symptom Check List-90 (SCL-90) questionnaire, Hospital Anxiety and Depression Scale (HADS) questionnaire and Social Support Rating Scale (SSRS) questionnaire were used to evaluate clinical features and psychological assessment, respectively, within 1 week before MRI (19–21).

### Image Acquisition

All MRI data were acquired on a SIEMENS SKYRA 3.0T magnetic resonance scanner (Siemens, Germany) in the Department of Radiology at the Union Hospital, Tongji Medical College, Huazhong University. All of the subjects were instructed to avoid caffeine or other similar substances before examination. They were kept in the supine position and asked to relax without falling asleep and to keep their eyes closed during scanning. Earplugs and a sponge pad were used to minimize scanner noise to protect the hearing and prevent head movement. Functional images were acquired with a single-shot gradient-recalled echo planar imaging sequence using the following parameters: time points = 240; slices = 60; slice thickness = 2.4 mm with no gaps, time of repetition (TR) = 2,000 ms; time of echo (TE) = 30 ms; flip angle (FA) = 90°; field of view (FOV) = 230 × 230 mm; and matrix size: 96 × 96; voxel size = 2.4 × 2.4 × 2.4 mm; bandwidth = 1796 Hz/Px, time = 8:13; parallel acquisition technique GRAPPA acceleration factor 2. We used a T1-weighted magnetization prepared rapid gradient echo (MP2RAGE) sequence, the following parameters: slices=176, slice thickness=1.0 mm with no gaps, TR = 5,000 ms; TE = 2.98 ms; FA = 9°, FOV = 256 × 240 mm, voxel size = 1 × 1 × 1 mm; bandwidth = 240 Hz/Px, time = 8:22parallel acquisition technique GRAPPA acceleration factor 3.

### Imaging Processing

The rs-fMRI data were preprocessed using DPARSF packages in SPM12 (https://www.fil.ion.ucl.ac.uk/spm) and DPABI software (http://rfmri.org/dpabi) (22). The preprocessing steps were as follows: (a) slice timing; (b) head movement correction (<2.0 mm or 2.0° in any direction); (c) spatial normalization by using diffeomorphic anatomical registration through exponentiated lie (DARTEL); (d) smoothing applied after ReHo analysis to avoid spurious connections; and (e) removal of linear trends. In addition, multiple linear regression models were used to regress the effects of white matter and cerebrospinal fluid signals on the processed resting-state data.

### Statistical Analysis

Clinical measurements and demographics were compared between groups using IBM SPSS Statistics 26.0 software. A two independent-samples T test and one-way analysis of variance (ANOVA) were used for normally distributed continuous variables such as age, body mass index (BMI), years of education and psychological assessment scores. Gender was calculated by Chi-square test. Pearson's correlation was applied to evaluate the relationship between the ReHo values of brain regions, clinical features and psychological assessment scores. All *P* values were two tailed, and *P* < 0.05 was considered statistically significant.

For ReHo, one-way-ANCOVA of the DPABI statistical module was utilized at each voxel to assess the main effect with a significance level of voxel *P* < 0.001 and cluster *P* < 0.01 (GRF corrected). Age, gender, mean FD Jenkinson were used as covariates. Pearson's linear correlation analyses were applied to assess the relationships among the ReHo, clinical features and psychological assessment scores in active and inactive CD patients (statistical significance level *P* < 0.05).

## Results

### Demographic and Clinical Characteristics

As shown in Table 1, the MRI data of 207 participants were included in the analysis. There were no significant differences observed in gender, age, education, BMI or duration among the three groups (*P* > 0.05). The CRP and ESR levels in the active CD group were higher than those in the inactive CD group, whereas the PLT levels between the two groups were not significantly different. Meanwhile, the IBDQ, HADS, SCL-90 and SSRS scores among the three groups were significant difference, whereas the active CD group and the inactive CD group were not significantly different. The active CD group exhibited the lowest IBDQ scores, SSRS scores, and HADS scores and the highest SCL-90 scores. The psychological assessment scores of the HC group were within the normal range, as shown in Table 2.

**Table 1:** Demographic and clinical features of all participants.

**Characteristics**	**HC (*n =* 92)**	**Active CD (*n =* 58)**	**Inactive CD (*n =* 57)**	***P* value**
Gender (M/F)	66:26	47:11	44:13	0.435^b^
Age (years)	28.65 ± 9.49	30.09 ± 11.23	32.56 ± 11.32	0.090^a^
Education (years)	17.40 ± 2.53	16.80 ± 2.11	16.9 ± 2.51	0.261^a^
BMI	19.98 ± 3.37	19.80 ± 3.68	20.00 ± 4.10	0.946^a^
Duration (years)	–	1.23 ± 1.11	1.67 ± 1.51	0.077^c^
CDAI	–	284.22 ± 38.23	86.51 ± 26.93	<0.001^c^^*^
SES-CD	–	13.86 ± 7.03	2.81 ± 1.74	<0.001^c^^*^
CRP	–	25.37 ± 30.04	10.15 ± 17.01	<0.001^c^^*^
ESR	–	21.90 ± 19.58	12.68 ± 14.51	0.012^c^^*^
PLT	–	300.83 ± 111.67	265.52 ± 109.77	0.700^c^
Montreal classification	–	–	–	–
L1:L2:L3:L4	–	14:7:33:4	12:6:36:3	0.920^b^
B1:B2:B3:P	–	14:18:26:29	21:12:24:23	0.379^b^

*The values are presented as mean values ± standard deviations*.

*HC, healthy controls; CD, Crohn's disease; BMI, Body Mass Index; CDAI, Crohn's disease Activity Index; SES-CD, Simple endoscopic score for Crohn's disease; CRP, C-reactive protein; ESR, erythrocyte sedimentation rate; PLT, platelet levels; L1, ileum; L2, colonic; L3, ileocolonic; L4, upper gastrointestinal tract disease; B1, on inflammatory and no penetrating; B2, inflammatory; B3, penetrating; P, perianal disease*.

a *P value for one-way ANOVA*.

b *P value for Chi-square test*.

c *P value for independent-sample T-test*.

* *Significant difference between active CD group and inactive CD group (P <0.05)*.

**Table 2:** Psychological assessment scores of all participants.

**Characteristics**	**HC (*n* = 92)**	**Active CD** **group (*n* = 58)**	**Inactive CD** **group (*n* = 57)**	***P* value**
IBDQ	–	–	–	-
Total	164.31 ± 32.65^*^^#^	122.16 ± 34.89	137.61 ± 50.56	<0.05
Bowel symptoms	64.22 ± 2.31^*^^#^	55.31 ± 7.64^&^	58 ± 6.89	<0.05
Systemic symptoms	29.00 ± 1.42^*^^#^	24.44 ± 4.30	23.69 ± 4.83	<0.05
Emotional function	77.00 ± 3.91^*^^#^	60.28 ± 9.78	62.69 ± 8.45	<0.05
Social impairment	35.42 ± 4.61^*^^#^	25.28 ± 5.54	23.81 ± 6.21	<0.05
HADS	–	–	–	-
Anxiety	9.33 ± 2.59^*^^#^	5.10 ± 3.47	5.40 ± 3.35	<0.05
Depression	9.14 ± 2.54^*^^#^	5.10 ± 3.97	4.89 ± 3.97	<0.05
SCL-90	–	–	–	-
Total	110.7 ± 19.52^*^^#^	134.25 ± 26.00	135.38 ± 39.75	<0.05
SOM	14.63 ± 3.81^*^^#^	16.69 ± 3.47	18.19 ± 6.21	<0.05
O-C	14.32 ± 3.99^*^^#^	16.56 ± 4.21	17.25 ± 5.20	<0.05
I-S	11.13 ± 2.13^*^^#^	14.06 ± 4.61	13.21 ± 5.08	<0.05
DEP	16.17 ± 3.53^*^^#^	21.94 ± 6.07	20.38 ± 6.09	<0.05
ANX	12.24 ± 2.49^*^^#^	14.31 ± 3.73	14.44 ± 4.37	<0.05
HOS	7.26 ± 1.98^*^^#^	9.38 ± 2.61	9.38 ± 4.21	<0.05
PHOB	7.74 ± 1.08^#^	8.25 ± 1.93	9.06 ± 3.04	<0.05
BIG	6.67 ± 1.19^*^^#^	7.97 ± 1.84	8.12 ± 3.69	<0.05
PSY	11.26 ± 1.89^*^^#^	13.34 ± 3.30	14.19 ± 4.84	<0.05
other	8.96 ± 2.15^*^^#^	11.75 ± 2.93	11.06 ± 2.67	<0.05
SSRS	–	–	–	-
Total	44.02 ± 1.35^*^^#^	30.76 ± 7.43^&^	33.65 ± 8.56	<0.05
Objective support	11.31 ± 1.56^*^^#^	8.64 ± 2.45	9.33 ± 3.05	<0.05
Subjective Support	22.05 ± 2.27^*^^#^	15.17 ± 5.37^&^	17.26 ± 6.01	<0.05
Availability	10.13 ± 0.96^*^^#^	6.95 ± 1.71	7.05 ± 1.84	<0.05

*Psychological assessment index among three groups based on one-way ANOVA. The values are presented as mean values ± standard deviations*.

*HC, healthy controls; CD, Crohn's disease; IBDQ, Inflammatory Bowel Disease Questionnaire; HADS, Hospital Anxiety and Depression Scores; SCL-90, Symptom check list-90; SOM, somatization; O-C, Obsessive-Compulsive; I-S, Interpersonal sensitivity; DEP, depression; ANX, anxiety; HOS, Hostility; PHOB, Phobic anxiety, BIG, Bigoted, PSY, Psychoticism; SSRS, Social Support Rating Scale*.

* *Indicates a significant difference between HC and active CD (P <0.05)*.

# *Indicates a significant difference between HC and inactive CD group (P <0.05)*.

& *Indicates a significant difference between active CD group and inactive CD group (P <0.05)*.

### ReHo Analysis

**Figure 2:**
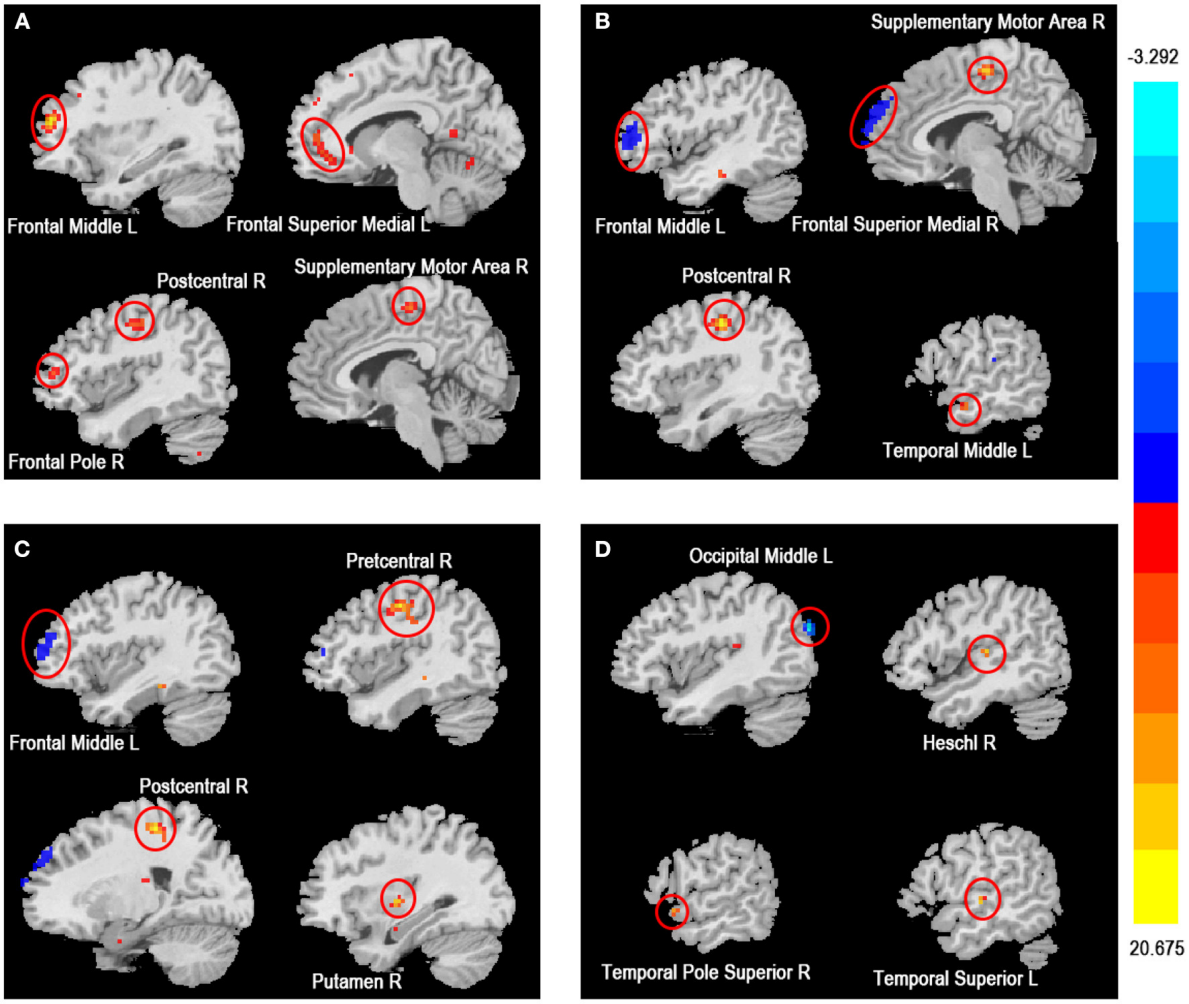
Brain regions with abnormal ReHo among three groups based on one-way ANOVA. Significant increased (red) and decreased (blue) ReHo values in among active CD, inactive CD, and HC groups (GRF corrected, voxel *p* < 0.001, cluster *p* < 0.01). The color bar represents the t-value. **(A)** The main effect analysis revealed significant differences in ReHo values of the left frontal middle, left frontal superior medial, right postcentral, right frontal pole and right supplementary motor area among the three groups. **(B)** The *post-hoc* analysis showed that the ReHo values of the right frontal superior medial, left frontal middle in the HC group were lower and the right postcentral, right supplementary motor area, and left temporal middle were higher than the ReHo values of the active CD group. **(C)** The *post-hoc* analysis showed that the ReHo values of the left frontal middle in the HC group was lower and the right precentral, right postcentral and right putamen were higher than the ReHo values of the inactive CD group. **(D)** The *post-hoc* analysis showed that the ReHo values of the left occipital middle in the active CD group was lower and the right heschl, right temporal pole superior and left temporal superior were higher than the ReHo values of the inactive CD group. CD, Crohn's disease (CD); ReHo, regional homogeneity; HC, healthy controls; L, left; R, right.

As shown in Table 3 and Figure 2, the ReHo values of the right frontal superior medial and left frontal middle in the active CD group were significantly higher than those in the HC group, whereas the ReHo values of the right postcentral, supplementary motor area and left temporal middle in the active CD group were significantly lower than those in the HC group (voxel *p* < 0.001, cluster *p* < 0.01, and two tailed, GRF correction). The ReHo values for the left frontal middle in the inactive CD group were significantly higher than those in the HC group, whereas the ReHo values of the right precentral, postcentral and putamen were significantly lower than those in the HC group (voxel *p* < 0.001, cluster *p* < 0.01, and two tailed, GRF correction). In active CD patients, the ReHo values of the left occipital middle were higher than those of inactive CD patients, whereas those of the right Heschl, temporal pole superior, and left temporal superior pole were lower (voxel *p* < 0.001, cluster *p* < 0.01, and two tailed, GRF correction).

**Table 3:** ReHo alterations of all participants.

**Main brain region**	**Hem**	**BA**	**MNI peak coordinate**			**Voxel size**	**t-value**
			**X**	**Y**	**Z**
**HC < active CD**	–	–	–	–	–	–	–
Frontal superior medial	R	10	6	69	9	91	−3.29
Frontal middle	L	46	−45	48	15	98	−3.29
**HC > Active CD**	–	–	–	–	–	–	-
Postcentral	R	3	39	−21	45	54	4.99
Supplementary motor area	R	4	6	−21	57	30	4.68
Temporal middle	L	21	−57	−6	−15	26	4.02
**HC < inactive CD**	–	–	–	–	–	–	-
Frontal middle	L	46	−39	51	9	121	−3.29
**HC > inactive CD**	–	–	–	–	–	–	-
Precentral	R	6	42	−12	45	36	4.35
Postcentral	R	3	21	−30	57	33	4.46
Putamen	R	48	33	−9	3	25	4.12
**Active CD < inactive CD**	–	–	–	–	–	–	-
Occipital middle	L	19	−42	−81	27	14	−2.61
**Active CD > inactive CD**	–	–	–	–	–	–	-
Heschl	R	48	48	−21	9	9	3.24
Temporal pole superior	R	38	60	12	−6	6	3.17
Temporal superior	L	48	−54	−15	0	6	3.21

*Brain regions exhibiting significant differences in ReHo values among HC group, active CD group and inactive CD group*.

*The results employed age, gender, FD Jenkinson as covariates. The statistical threshold was set at voxel p <0.001, cluster p <0.01, and two tailed (GRF correction)*.

*HC, healthy controls; CD, Crohn's disease; Hem, hemisphere; BA, Brodmann area; L, left; R, right*.

### Correlation Among ReHo, Clinical Features and Psychological Assessment Scores

**Figure 3:**
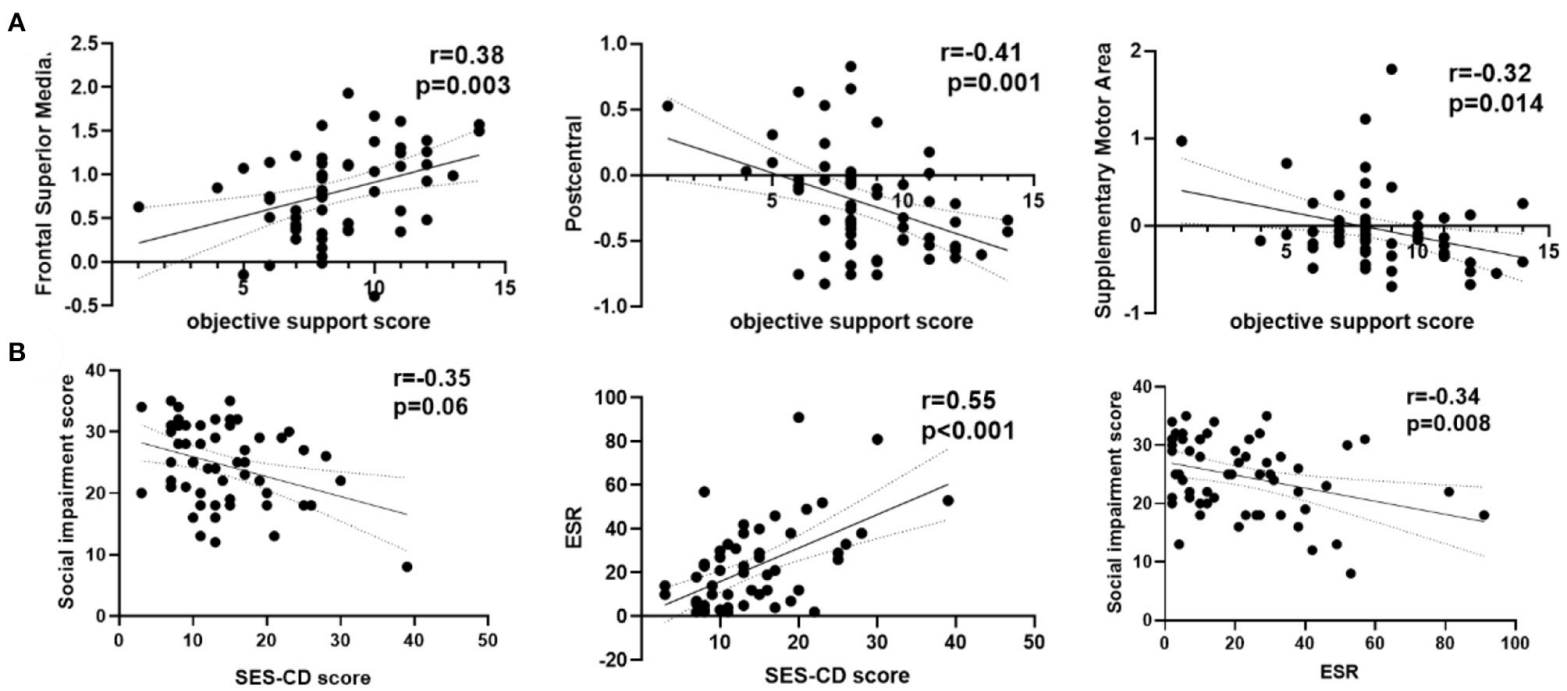
Correlation analysis among ReHo, clinical features and psychological assessment scores in active CD group. **(A)** The ReHo values of the right frontal superior medial were positively and the right postcentral, right supplementary motor area in active CD group were negatively correlated with the objective support scores. **(B)** The social impairment score were negatively and ESR positively correlated with the SES-CD scores. CD, Crohn's disease; SES-CD, Simple endoscopic score for Crohn's disease; ESR, erythrocyte sedimentation rate; r, correlation coefficient.

**Figure 4:**
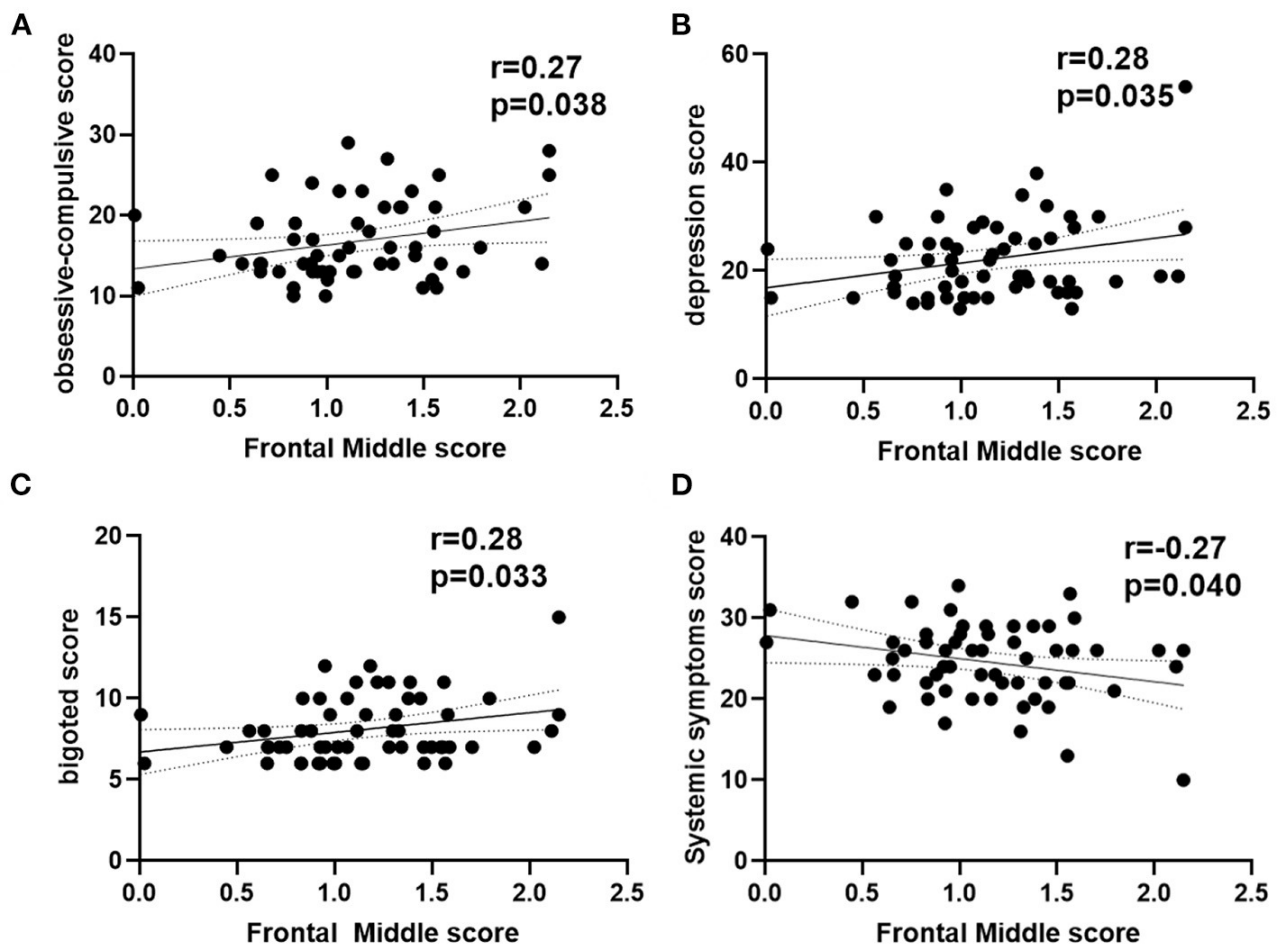
Correlation analysis between ReHo in left frontal middle score with psychological assessment score in active CD group. **(A)** The ReHo in left frontal middle was positively with the obsessive-compulsive scores. **(B)** The ReHo in left frontal middle was positively with the depression scores. **(C)** The ReHo in left frontal middle was positively with the bigoted scores. **(D)** The ReHo in left frontal middle was negatively with the systemic symptoms scores. CD, Crohn's disease; ReHo, regional homogeneity; r, correlation coefficient.

As shown in Figure 3, the ReHo of the right frontal superior medial brain regions was positively correlated with the psychological assessment of objective support in active CD patients, and negatively correlated with right postcentral and supplementary motor area brain regions, respectively (r = 0.38,−0.41, −0.32, *P* = 0.001, 0.014, 0.003), but not significantly correlated with the ReHo values in the left frontal middle and temporal middle brain regions (r = 0.07, −0.11, *P* = 0.671, 0.430). And the ESR was positively correlated with the SES-CD scores and negatively correlated with the psychological assessment of social impairment score (r = 0.55, −0.34, *P*<0.001, 0.008). As shown in Figure 4, the ReHo values of the left frontal middle brain regions were positively correlated with the obsessive-compulsive, depression, and bigoted scores, and negatively correlated with the systemic symptoms score in these CD patients respectively (r = 0.27, 0.28, 0.28, −0.27, *P* = 0.038, 0.035, 0.034, 0.004).

## Discussion

This is a study to research the functional alterations of regional brain areas in active and inactive CD patients with psychological assessment disorders based on ReHo. The results of this study showed that active CD patients had more severe psychological assessment scores and intestinal clinical features than in inactive patients, which correlated with significantly increased ReHo in the right frontal superior medial and left frontal middle and decreased ReHo in the right postcentral, supplementary motor area, and left temporal middle. Correlation analysis revealed that these brain regions were associated with psychological assessment scores.

In this study, we found that the active CD patients had the most severe psychological assessment scores. The social impairment, emotional, depression, anxiety and other psychological assessment scores were closed to the cut-offs for remission psychopathology patients. This indicates that disease activity severely affects the quality of life, psychological state and social relations of CD patients, which is consistently with Leone and Chavarria et al. previous results (23, 24).

The ReHo values in the frontal middle gyrus and postcentral gyrus were both changed in the active and inactive CD groups compared with the HC group, which suggested that these brain regions were involved in the occurrence of CD. The postcentral gyrus of the cerebral cortex has a sensory area that explains various sensory stimuli. Therefore, functional abnormalities of the frontal middle and postcentral gyrus may also be a neurobiological mechanism for the occurrence of CD.

In inactive CD patients, the ReHo values in the left frontal middle, right precentral, postcentral and putamen also showed changes compared with the HC group. Similar changes were found for ulcerative colitis (25). In inactive CD patients, the ReHo values in the superior frontal gyrus, putamen, anterior cingulate and supplementary motor areas were altered in other study (15, 26, 27). In addition, Bao et al. found that the gray matter volumes in CD patients were changed in that brain region (8), which suggested that CD patients have abnormal brain activity and morphology. We also found that the ReHo values of brain regions in the right frontal superior medial, left frontal middle, right postcentral, supplementary motor area and left temporal middle in active CD patients showed differences compared with the HC group.

Compared with inactive CD patients, the ReHo values of active CD patients in left occipital middle and temporal superior regions and in the right heschl and temporal pole superior regions were different. This is the first evidence of alterations in ReHo values in active CD patients. These results suggest a different alteration in the ReHo values of spontaneous BOLD signals between active and inactive CD patients. This may indicate that brain activity and disease activity are associated in CD patients.

In this study, compared with HC group, we also discovered added brain regions in active CD patients. This may be due to the sustained disease puzzle signals input to the brain when they are in active stage. The frontal superior medial region is a significant brain region for cognitive function control that may lead to increased or enhanced thoughts, memories or emotion effects and can indirectly or directly modulate the processing of intestinal stimuli (28, 29). The supplementary motor area is part of the primary motor cortex and is mainly involved in voluntary motor control of the trunk muscles (30, 31). The role of the temporal middle gyrus is to process speech and hearing. This indicates that those brain regions are adaptations to decreased nociceptive input or represent a preexisting vulnerability to experiencing greater activity disease. In these regions, the ReHo values were not significantly different between the inactive CD patients and the HC group. The noxious stimulus signals of the intestinal inflammation can be passed to the brain through gastrointestinal afferent sensory fibers and vagal afferent fibers of the spinal nerves (32, 33). Some studies have confirmed that proinflammatory cytokines and chemokines play a significant role in communication between the systemic system and the brain during the active stage (34, 35). Therefore, it is important to consider frontal superior medial gyrus, supplementary motor area gyrus and temporal middle gyrus dysfunction as potential pathophysiologic mechanisms that cause psychological disorders.

In our study, we also performed correlation analysis to attempt to identify the relationship between these changes in brain regions with clinical intestinal features and psychological assessment indicators. An increased objective support score in active CD patients with increased ReHo values of the right frontal superior medial and reduced ReHo values of the right postcentral and supplementary motor area brain regions indicate that the neural activities in these brain regions may have alternating synchronization and coordination. The psychological assessment index of social impairment score had significant negative correlations with the SES-CD scores and ESR. This suggests that psychological assessment alterations might be secondary to intestinal disease in CD patients. The ReHo values of the frontal middle brain regions were positively correlated with the psychological assessment indices of the obsessive-compulsive, depression, and bigoted scores and negatively correlated with the systemic symptoms score in these CD patients. CD is a systemic disorder (36) in which the lesions are not limited to the gastrointestinal tract but involve an imbalanced homeostasis of the autonomic nervous system environment. Abnormalities in this brain region indicate an imbalance or dysfunction of internal environment homeostasis.

This study indicates that CD patients often have psychological disorders, and clinical intestinal symptoms and psychological disorder symptoms affect each other. Therefore, when treating patients with CD, physicians should not only pay attention to clinical intestinal symptoms but also consider emotional symptoms to maximize medical management and improve quality of life. The results of this research are not completely consistent with previous studies for several possible reasons. First, we used different subjects. In this study, we selected active and inactive CD patients, whereas in previous studies, the patients had inactive CD. Second, psychological state assessment includes not only anxiety and depression but also other emotions, interpersonal communication, and social support.

Several limitations should be considered in this study. First, this is a cross-sectional and single-center study. We could not confirm whether the abnormal changes in ReHo values in CD patients occurred before or after the onset of the disease. Thus, longitudinal research should be conducted to establish a more robust connection between spontaneous BOLD signals and psychological and disease activity in CD patients in the future. Second, multidimensional intestinal performance evaluation of imaging manifestations, including CT or MR, is also significant in CD patients and should be addressed in future studies. Finally, the voxel size of these brain regions that the ReHo value of active CD patients were higher than the inactive CD patients is small. Although it has been rigorously corrected, there is still the possibility of false positives. In the future study, we will include more CD patients to improve the reliability of our results.

In conclusion, the present study showed that active CD patients have more severe intestinal clinical features. Furthermore, alterations in ReHo in the frontal, putamen, postcentral, supplementary motor area and temporal regions may play a significant role in the neural basis of CD with psychological disorders. These findings can help us to better understand the brain-gut axis interaction and provide an insight to facilitate the development of new therapies in the future.

## Data Availability Statement

The raw data supporting the conclusions of this article will be made available by the authors, without undue reservation.

## Ethics Statement

The studies involving human participants were reviewed and approved by the Institutional Ethics Committee of Tongji Medical College of Huazhong University of Science and Technology. The patients/participants provided their written informed consent to participate in this study.

## Author Contributions

MH, YZ, and PH: study design. MH, YZ, WF, JL, LZ, XL, PL, LW, and QS: analysis and interpretation of data. MH and YZ: drafting of the manuscript. MH, YZ, PL, JL, XL, and PH: critical revision of the manuscript. All authors: approval of the final version for submission.

## Funding

This work was financially supported by the National Natural Science Foundation of China (81873895 to PH).

## Conflict of Interest

The authors declare that the research was conducted in the absence of any commercial or financial relationships that could be construed as a potential conflict of interest.

## Publisher's Note

All claims expressed in this article are solely those of the authors and do not necessarily represent those of their affiliated organizations, or those of the publisher, the editors and the reviewers. Any product that may be evaluated in this article, or claim that may be made by its manufacturer, is not guaranteed or endorsed by the publisher.

## References

[1] Mak WY Zhao M Ng SC Burisch J. The epidemiology of inflammatory bowel disease: East meets west. J Gastroenterol Hepatol. (2020) 35:380–9. 10.1111/jgh.14872 31596960

[2] Sands BE. From symptom to diagnosis: clinical distinctions among various forms of intestinal inflammation. Gastroenterology. (2004) 126:1518–32. 10.1053/j.gastro.2004.02.072 15168364

[3] Baumgart DC Carding SR. Gastroenterology 1 - Inflammatory bowel disease: cause and immunobiology. Lancet. (2007) 369:1627–40. 10.1016/S0140-6736(07)60750-8 17499605

[4] Ng SC Tang W Ching JY Wong M Chow CM Hui AJ. Incidence and phenotype of inflammatory bowel disease based on results from the Asia-pacific Crohn's and colitis epidemiology study. Gastroenterology. (2013) 145:158–65.e2. 10.1053/j.gastro.2013.04.007 23583432

[5] Ng SC Shi HY Hamidi N Underwood FE Tang W Benchimol EI. Worldwide incidence and prevalence of inflammatory bowel disease in the 21st century: a systematic review of population-based studies. Lancet. (2017) 390:2769–78. 10.1016/S0140-6736(17)32448-0 29050646

[6] Lv H Jin M Zhang H Chen X Wu M Guo M. Increasing newly diagnosed inflammatory bowel disease and improving prognosis in China: a 30-year retrospective study from a single centre. BMC Gastroenterol. (2020) 20:377. 10.1186/s12876-020-01527-1 33183228 PMC7659043

[7] Høivik ML Moum B Solberg IC Henriksen M Cvancarova M Bernklev T. Work disability in inflammatory bowel disease patients 10 years after disease onset: results from the IBSEN Study. Gut. (2013) 62:368–75. 10.1136/gutjnl-2012-302311 22717453

[8] Bao CH Liu P Liu HR Wu LY Shi Y Chen WF. Alterations in brain grey matter structures in patients with crohn's disease and their correlation with psychological distress. J Crohns Colitis. (2015) 9:532–40. 10.1093/ecco-jcc/jjv057 25895879

[9] Agostini A Ballotta D Righi S Moretti M Bertani A Scarcelli A. Stress and brain functional changes in patients with Crohn's disease: A functional magnetic resonance imaging study. Neurogastroenterol Motil. (2017) 29:1–10. 10.1111/nmo.13108 28560758

[10] Gracie DJ Guthrie EA Hamlin PJ Ford AC. Bi-directionality of brain-gut interactions in patients with inflammatory bowel disease. Gastroenterology. (2018). 154:1635–46 e3. 10.1053/j.gastro.2018.01.027 29366841

[11] Goodhand JR Wahed M Mawdsley JE Farmer AD Aziz Q Rampton DS. Mood disorders in inflammatory bowel disease: relation to diagnosis, disease activity, perceived stress, and other factors. Inflamm Bowel Dis. (2012) 18:2301–9. 10.1002/ibd.22916 22359369

[12] Gao X Tang Y Lei N Luo Y Chen P Liang C. Symptoms of anxiety/depression is associated with more aggressive inflammatory bowel disease. Sci Rep. (2021) 11:1440. 10.1038/s41598-021-81213-8 33446900 PMC7809475

[13] Zang Y Jiang T Lu Y He Y Tian L. Regional homogeneity approach to fMRI data analysis. Neuroimage. (2004) 22:394–400. 10.1016/j.neuroimage.2003.12.030 15110032

[14] Bao CH Liu P Liu HR Wu LY Jin XM Wang SY. Differences in regional homogeneity between patients with Crohn's disease with and without abdominal pain revealed by resting-state functional magnetic resonance imaging. Pain. (2016) 157:1037–44. 10.1097/j.pain.0000000000000479 26761381 PMC4969077

[15] Thomann AK Schmitgen MM Kmuche D Ebert MP Thomann PA Szabo K. Exploring joint patterns of brain structure and function in inflammatory bowel diseases using multimodal data fusion. Neurogastroenterol Motil. (2021) 33:e14078. 10.1111/nmo.14078 33368950

[16] May A. Chronic pain may change the structure of the brain. Pain. (2008) 137:7–15. 10.1016/j.pain.2008.02.034 18410991

[17] Best WR Becktel JM Singleton JW. Rederived values of the eight coefficients of the Crohn's Disease Activity Index (CDAI). Gastroenterology. (1979) 77:843–6. 10.1016/0016-5085(79)90384-6 467941

[18] Sostegni R Daperno M Scaglione N Lavagna A Rocca R Pera A. Review article: Crohn's disease: monitoring disease activity. Aliment Pharmacol Ther. (2003) 17 Suppl 2:11–7. 10.1046/j.1365-2036.17.s2.17.x 12786607

[19] Irvine EJ Feagan B Rochon J Archambault A Fedorak RN Groll A. Quality of life: a valid and reliable measure of therapeutic efficacy in the treatment of inflammatory bowel disease. Canadian Crohn's relapse prevention trial study group. Gastroenterology. (1994) 106:287–96. 10.1016/0016-5085(94)90585-1 8299896

[20] Schmitz N Hartkamp N Kiuse J Franke GH Reister G Tress W. The Symptom Check-List-90-R (SCL-90-R): a German validation study. Qual Life Res. (2000) 9:185–93. 10.1023/A:1008931926181 10983482

[21] Melges FT Fougerousse CE Jr. Time sense, emotions, and acute mental illness. J Psychiatr Res. (1966) 4:127–39. 10.1016/0022-3956(66)90025-2 20034165

[22] Chao-Gan Y Yu-Feng ZDPARSF. A MATLAB toolbox for “pipeline” data analysis of resting-state fMRI. Front Syst Neurosci. (2010) 4:13. 10.3389/fnsys.2010.00013 20577591 PMC2889691

[23] Chavarría C Casanova MJ Chaparro M Barreiro-de Acosta M Ezquiaga E Bujanda L. Prevalence and factors associated with fatigue in patients with inflammatory bowel disease: a multicentre study. J Crohns Colitis. (2019) 13:996–1002. 10.1093/ecco-jcc/jjz024 30721954

[24] Leone D Gilardi D Corrò BE Menichetti J Vegni E Correale C. Psychological characteristics of inflammatory bowel disease patients: a comparison between active and nonactive patients. Inflamm Bowel Dis. (2019) 25:1399–407. 10.1093/ibd/izy400 30689871

[25] Agostini A Filippini N Cevolani D Agati R Leoni C Tambasco R. Brain functional changes in patients with ulcerative colitis: a functional magnetic resonance imaging study on emotional processing. Inflamm Bowel Dis. (2011) 17:1769–77. 10.1002/ibd.21549 21744432

[26] Li L Ma J Xu JG Zheng YL Xie Q Rong L. Brain functional changes in patients with Crohn's disease: A resting-state fMRI study. Brain Behav. (2021) 11:e2243. 10.1002/brb3.2243 34124857 PMC8413760

[27] Hou J Mohanty R Nair VA Dodd K Beniwal-Patel P Saha S. Alterations in resting-state functional connectivity in patients with Crohn's disease in remission. Sci Rep. (2019) 9:7412. 10.1038/s41598-019-43878-0 31092855 PMC6520362

[28] Agostini A Filippini N Benuzzi F Bertani A Scarcelli A Leoni C. Functional magnetic resonance imaging study reveals differences in the habituation to psychological stress in patients with Crohn's disease versus healthy controls. J Behav Med. (2013) 36:477–87. 10.1007/s10865-012-9441-1 22752251

[29] Grundy D. Neuroanatomy of visceral nociception: vagal and splanchnic afferent. Gut. (2002) 51 Suppl 1:i2–5. 10.1136/gut.51.suppl_1.i2 12077054 PMC1867719

[30] Butovsky O Weiner HL. Microglial signatures and their role in health and disease. #N/A. (2018) 19:622–35. 10.1038/s41583-018-0057-5 30206328 PMC7255106

[31] Rivest S. Molecular insights on the cerebral innate immune system. #N/A. (2003) 17:13–9. 10.1016/S0889-1591(02)00055-7 12615045

[32] Spencer NJ Hu H. Enteric nervous system: sensory transduction, neural circuits and gastrointestinal motility. Nat Rev Gastroenterol Hepatol. (2020) 17:338–51. 10.1038/s41575-020-0271-2 32152479 PMC7474470

[33] Thomann AK Griebe M Thomann PA Hirjak D Ebert MP Szabo K. Intrinsic neural network dysfunction in quiescent Crohn's Disease. Sci Rep. (2017) 7:11579. 10.1038/s41598-017-11792-y 28912568 PMC5599642

[34] Liu P Li R Bao C Wei Y Fan Y Liu Y. Altered topological patterns of brain functional networks in Crohn's disease. Brain Imaging Behav. (2018) 12:1466–78. 10.1007/s11682-017-9814-8 29297154

[35] Petra AI Panagiotidou S Hatziagelaki E Stewart JM Conti P Theoharides TC. Gut-microbiota-brain axis and its effect on neuropsychiatric disorders with suspected immune dysregulation. Clin Ther. (2015) 37:984–95. 10.1016/j.clinthera.2015.04.002 26046241 PMC4458706

[36] Dantzer R O'Connor JC Freund GG Johnson RW Kelley KW. From inflammation to sickness and depression: when the immune system subjugates the brain. Nat Rev Neurosci. (2008) 9:46–56. 10.1038/nrn2297 18073775 PMC2919277

